# Emerging trends in modeling human liver disease *in vitro*

**DOI:** 10.1063/1.5119090

**Published:** 2019-12-24

**Authors:** Gregory H. Underhill, Salman R. Khetani

**Affiliations:** 1 Department of Bioengineering, University of Illinois at Urbana-Champaign, Urbana, Illinois 61801, USA; 2 Department of Bioengineering, University of Illinois at Chicago, Chicago, Illinois 60607, USA

## Abstract

The liver executes 500+ functions, such as protein synthesis, xenobiotic metabolism, bile production, and metabolism of carbohydrates/fats/proteins. Such functions can be severely degraded by drug-induced liver injury, nonalcoholic fatty liver disease, hepatitis B and viral infections, and hepatocellular carcinoma. These liver diseases, which represent a significant global health burden, are the subject of novel drug discovery by the pharmaceutical industry via the use of *in vitro* models of the human liver, given significant species-specific differences in disease profiles and drug outcomes. Isolated primary human hepatocytes (PHHs) are a physiologically relevant cell source to construct such models; however, these cells display a rapid decline in the phenotypic function within conventional 2-dimensional monocultures. To address such a limitation, several engineered platforms have been developed such as high-throughput cellular microarrays, micropatterned cocultures, self-assembled spheroids, bioprinted tissues, and perfusion devices; many of these platforms are being used to coculture PHHs with liver nonparenchymal cells to model complex cell cross talk in liver pathophysiology. In this perspective, we focus on the utility of representative platforms for mimicking key features of liver dysfunction in the context of chronic liver diseases and liver cancer. We further discuss pending issues that will need to be addressed in this field moving forward. Collectively, these *in vitro* liver disease models are being increasingly applied toward the development of new therapeutics that display an optimal balance of safety and efficacy, with a focus on expediting development, reducing high costs, and preventing harm to patients.

NOMENCLATURE

CCC: cholangiocellular carcinoma
CYP450: cytochrome P450
DILI: drug-induced liver injury
ECM: extracellular matrix
FDA: Food and Drug Administration
FFA: free fatty acids
HBV: hepatitis B virus
HCC: hepatocellular carcinoma
HCV: hepatitis C virus
HSCs: hepatic stellate cells
HUVECs: human umbilical vein endothelial cells
iHeps: induced pluripotent stem cell-derived human hepatocytelike cells
iPSCs: induced pluripotent stem cells
JAK: janus kinase
KCs: Kupffer cells
LSECs: liver sinusoidal endothelial cells
MOI: multiplicity of infection
MPCCs: micropatterned cocultures
MPTCs: micropatterned tr-cultures
NAFLD: nonalcoholic fatty liver disease
NPCs: nonparenchymal cells
PDMS: polydimethylsiloxane-siloxane
PDX: patient-derived xenograft
PEG: polyethylene glycol
PHHs: primary human hepatocytes

## INTRODUCTION

The liver is the largest internal organ in the body and executes well over 500 functions, including (a) the metabolism of carbohydrates, fats, and proteins; (b) production of serum proteins such as albumin, transferrin, and clotting factors; (c) biotransformation of lipophilic pharmaceutical and industrial compounds into water-soluble metabolites that can be excreted from the body; and (d) production of bile that aids in the digestion of fats and fat-soluble vitamins in the intestine. These critical functions can be compromised by drug-induced liver injury (DILI) as well as several liver diseases such as nonalcoholic fatty liver disease (NAFLD), infection with hepatitis B and C viruses (HBV and HCV, respectively), and hepatocellular carcinoma (HCC). Many of these diseases represent significant global health burdens. For instance, DILI is a leading cause of preclinical and clinical drug failures, black-box warnings and withdrawals of marketed drugs, and acute liver failures; overall, DILI has been linked to ∼1000 marketed drugs^1,2^ NAFLD affects almost a third of the US population, and individuals with either type 2 diabetes mellitus or obesity are disproportionately affected;^3^ the number of cases with NAFLD is expected to rise from 83.1 million people in 2015 to 100.9 million in 2030.^4^ Finally, HBV and HCV infect the livers of more than 350 million people globally.^5^

A common feature of the liver diseases discussed above is that they increase patient risk to the development of liver fibrosis, cirrhosis, and ultimately HCC, which are the most common primary liver malignancy and the second leading cause of cancer-related deaths worldwide.^6^ Once patients develop decompensated cirrhosis and/or HCC, orthotopic liver transplantation is the only option to significantly extend their lives; however, there is a severe shortage of donor organs and the list of patients waiting for a liver transplant continues to grow. Halting disease progression prior to the initiation of cirrhosis and HCC is the critical goal for pharmaceutical development. While the latest drugs for HCV are highly effective (>90% cure rates), there is no vaccine available; additionally, the current drug therapies are very expensive (∼$1K per pill and ∼$84K for a 12-week treatment regimen^7^) to be disseminated globally outside of the industrialized nations. For HBV, current drugs are not curative and lifetime drug therapy is required. Finally, there are currently no drugs approved by the US Food and Drug Administration (FDA) for NAFLD, while surgical resection or liver transplantation is the best option for long-term survival in HCC patients as drug therapies have not shown to provide the survival advantage beyond a few weeks. Therefore, there is active interest in the pharmaceutical industry to develop novel drug therapies for the above-discussed liver diseases.

The FDA requires preclinical drug testing in one rodent and one nonrodent animal species to mitigate the risk of adverse effects in humans. However, it is now clear via several high-profile clinical drug failures that animal models do not completely suffice to mitigate the risk of DILI, likely due to significant differences across species in drug metabolism pathways.^8^ Additionally, testing drugs in isogenic strains of rodents does not adequately capture the risk factors in humans such as pre-existing disease, age, gender, nutritional status, comedication, and genetic predisposition. Therefore, animal models are less than 50% predictive of human DILI.^1,9^ Rodent models also do not suffice for mimicking key features and progression of liver diseases. For instance, HBV and HCV do not infect rodents unless the livers are “humanized” with transplantation of human liver cells under an injury background;^10^ however, humanized rodent models are very expensive and labor-intensive to create and present the challenge of a humanized liver interacting with other rodent organs. Similarly, testing HBV drugs on the chimpanzee is prohibitively expensive, severely restricted in the US and Europe, and does not fully mimic human HBV pathogenesis. NAFLD-like phenotypes can be created in rodent models via a combination of genetic, chemical, or nutritional conditions; however, none of these approaches entirely mimics the human condition.^11^ The mouse model of NAFLD that most closely recapitulates human NAFLD is the “Diet-induced Animal Model of Nonalcoholic Fatty Liver Disease” (DIAMOND) in which mice of a specific genetic strain which are fed a high fat and fructose diet develop progressive stages of NAFLD and end with HCC.^11,12^ However, ultimately, this model is limited to the specific genetic strain of mouse, takes months to recapitulate phenotypic features, and is too slow and expensive for high-throughput drug discovery.

Owing to the aforementioned major limitations with animal models, there has been considerable interest in the regulatory and pharmaceutical communities to adopt *in vitro* models of the human liver, which can be employed across all stages of drug development for investigating drug metabolism and toxicity, and to develop novel drug therapies against liver diseases. The ultimate goal of utilizing *in vitro* tissue models is to reduce the astronomical cost and time required for successful drug development (∼$3–5B and 12–15 years to bring 1 drug to the market^13,14^) as well as prevent harm to patients.

In the native liver, primary human hepatocytes (PHHs) represent nearly 80% of liver volume (60% of the total cell population) and execute a majority of liver functions. These cells are also surrounded by resident liver nonparenchymal cells (NPCs) that represent ∼6.5% of liver volume and 40% of the total population of cells.^15^ Liver NPCs include liver sinusoidal endothelial cells (LSECs), hepatic stellate cells (HSCs), and Kupffer cells/macrophages (KCs), which modulate hepatic functions in physiology and disease via paracrine and juxtacrine interactions. Additionally, the cholangiocytes form the lining of the bile ducts in the liver, which constitutes a separate flow system from the blood flow through the liver sinusoids. All these cell types can now be isolated via established protocols from human livers that are rejected for transplantation into patients;^16^ furthermore, cryopreserved human liver cells are now available commercially from several companies (e.g., Lonza, Bio-IVT, and Thermo-Fisher). It is preferable to use primary human liver cells instead of cancerous cell lines since the latter are known to display abnormal proliferation and very low liver-specific functions (e.g., drug metabolism enzymes).^17^ However, culture of PHHs and other liver cell types in conventional 2D monolayers leads to a decline in phenotypic functions within hours.^16^ The loss of functions can be slowed down but not prevented beyond a few days by extracellular matrix (ECM) manipulations such as overlaying a PHH monolayer with a protein gel made of either collagen or Matrigel™.^18^ More recently, the inclusion of 5 chemicals that modulate key hepatic pathways in the culture medium was shown to prolong some hepatic functions for 30 days and enable HBV infection of primary human hepatocytes *in vitro.*^19^ However, how the inclusion of these synthetic chemicals affects the physiology of the cell in unexpected ways remains to be determined. Furthermore, inclusion of chemicals in the culture medium during compound screening is typically problematic for drug development efforts in our experience.

In contrast to rapidly declining conventional PHH monocultures, several bioengineered culture strategies have been developed to enable precise control of culture conditions, which has led to relative phenotypic stability of human liver cells for several weeks *in vitro*. In this perspective, we discuss design features and validation of key bioengineered human liver platforms that have been utilized for modeling liver disease with special focus on HBV/HCV, NAFLD, and liver cancer since these represent the majority of global burden of liver disease over the next few decades. We also highlight pending issues and emerging trends in this field. While the evaluation of drug metabolism and toxicity represents a major application of human liver models and the first point of entry for such models into the marketplace, we refer the readers to other articles that comprehensively discuss validation datasets in this area;^20,21^ nonetheless, most of the platforms we discuss in this review have been utilized for drug metabolism and toxicity studies.

## HEPATITIS B AND C VIRUSES

HCV and HBV chronically infect the livers of 130–170 million and 400 million people worldwide, respectively. HCV/HBV only infects hepatocytes of humans, chimpanzees, tree shrews (tupaia belangeri, HCV), and woodchuck (HBV). Although the cost of direct acting antiviral drug therapies for HCV is decreasing, the global dissemination of these therapies remains challenging, and further, prophylactic treatments for HCV are not available. While HBV can be suppressed using nucleoside/nucleotide inhibitors, these therapies are not curative due to the stable nature of HBV episomal DNA, which necessitates lifetime drug therapy. Thus, since testing HCV/HBV drugs on chimpanzees is cost prohibitive and restricted in the US/Europe and tree shrew is not a standard preclinical drug screening animal, there is a need for robust human-relevant model systems for developing novel therapeutics that are curative via the disruption of either the virus lifecycle and/or its interaction with the host. While transformed hepatic cell lines are capable of supporting the entire lifecycle of HCV/HBV, they have uncontrolled proliferation and altered host responses to infection.^22,23^ Additionally, cell lines display severely low drug metabolism capacity,^24^ which can confound the results of a *de novo* drug screen. On the other hand, conventional 2D PHH monocultures can be infected with both viruses and are a more physiological representation of infection than transformed cell lines;^25,26^ however, maintaining chronic (weeks) infection in such monocultures across multiple donors is very challenging and there is a rapid decline in drug metabolism enzyme (e.g., cytochrome P450 or CYP) activities^27^ that do not fully mimic drug metabolism in the liver.

Unlike randomly distributing cells on a substrate, which do not allow for the precise control over cell-cell interactions that affect cell phenotype, photolithographic and soft-lithographic techniques have been used to fabricate micropatterned cocultures (MPCCs) of PHHs organized onto circular collagen-coated domains of empirically optimized dimensions and surrounded by 3T3-J2 murine embryonic fibroblasts.^27^ Precisely tuning the homotypic and heterotypic cell-cell interactions in MPCCs allows for relative stability of hepatic functions for up to 6 weeks *in vitro* in industry-standard multiwell plates without the need for any fluid perfusion.^28–30^ Interestingly, the 3T3-J2 fibroblasts induce higher functions in hepatocytes than other 3T3 subclones,^31^ LSEC,^32^ KCs,^33^ and HSCs,^34^ likely due to the expression of liverlike molecules such as T-cadherin^35^ and decorin^31^ by the 3T3-J2 fibroblasts; nonetheless, the above-mentioned liver-derived NPCs, when cultured with the fibroblasts, are still able to interact with stabilized PHHs for modeling specific disease scenarios as we discuss in subsequent paragraphs. In addition to primary cells, the MPCC platform can further mature adultlike functions in induced pluripotent stem cell-derived human hepatocytelike cells (iHeps),^36^ which afford the opportunity to sustainably evaluate disease and drug responses across diverse genetic backgrounds.

**FIG. 1.**
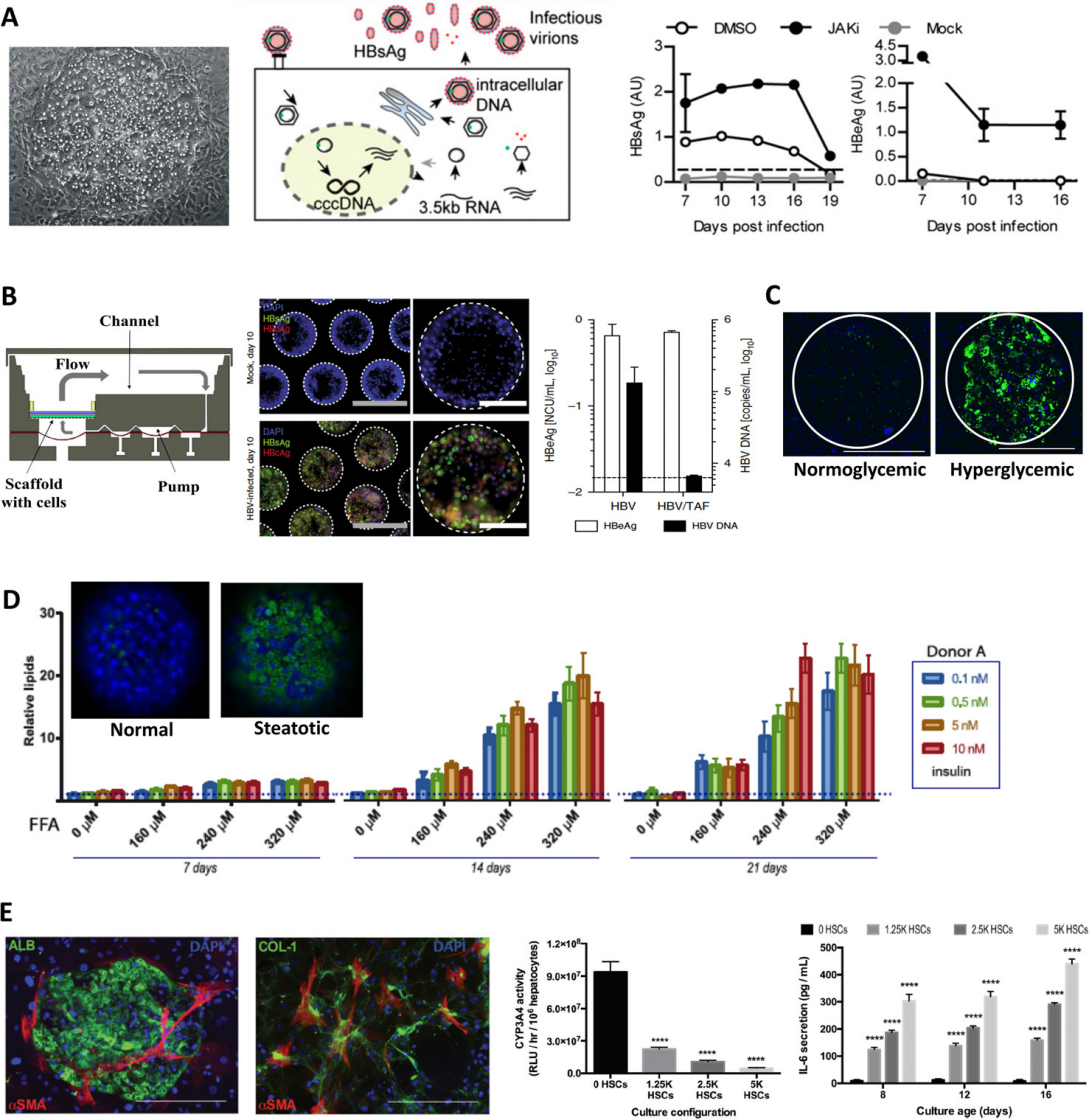
*In vitro* human liver models of HBV and NAFLD. (a) Infection of MPCCs with HBV.^38^ Left to right: Phase contrast image of a PHH island surrounded by 3T3-J2 fibroblasts. Schematic of HBV infection and lifecycle inside a hepatocyte. Viral antigens and cccDNA are detectable in MPCCs at higher levels when the cultures are incubated with a small molecule inhibitor of JAK. Reprinted with permission from Shlomai *et al.*, Proc. Natl. Acad. Sci. **111**(33), 12193 (2014). Copyright 2014 National Academy of Sciences. (b) HBV infection in the LiverChip.^40^ Left: Bioreactor cross section schematic showing the fluid flow direction and polycarbonate filter on which cells attach in spheroidal structures. Middle: HBV antigens (HBV “e” antigen or HBeAg in green; HBV “core” antigen or HBcAg in red; nuclei are in blue) were present in the self-assembled spheroids within the chip following infection with HBV. Scale bars: white (200 *μ*m), gray (100 *μ*m). Right: HBeAg and HBV DNA secretion in supernatants of HBV-infected cultures in the LiverChip ± treatment with HBV drug, tenofovir alafenamide (TAF, 1 *μ*M, 10 day treatment). Reprinted with permission from Ortega-Prieto *et al.*, Nat. Commun. **9**(1), 682 (2018); Copyright 2018 Author(s), licensed under a Creative Commons Attribution (CC BY) license. (c) PHHs in MPCCs treated with a hyperglycemic culture medium for 18 days become steatotic (green: Nile red, stains neutral lipids).^52^ Scale bars are 400 *μ*m. Reprinted with permission from Davidson *et al.*, Sci. Rep. **6**, 28178 (2016). Copyright 2016 Authors, licensed under a Creative Commons Attribution (CC BY) license. (d) Self-assembled PHH spheroids created using ultralow attachment plates can be made steatotic (green: Nile red stain for neutral lipids; blue: nuclei stained with Hoechst 33342) by incubating over time with varying concentrations of insulin and free fatty acids (FFA).^54^ with permission Kozyra *et al.*, Sci. Rep. **8**(1), 14297 (2018). Copyright 2018 Author(s), licensed under a Creative Commons Attribution (CC BY) license. (e) MPTCs containing PHHs, 3T3-J2 fibroblasts, and activated (fibrogenic) HSCs.^34^ Left to right: Fluorescence images showing albumin (ALB) positive PHHs, alpha smooth muscle actin (alpha-SMA) positive activated HSCs, and collagen (COL-1) deposition by the HSCs as in liver fibrosis. CYP3A4 enzyme activity in PHHs is downregulated with the addition of increasing numbers of HSCs in the MPTC model. Higher levels of inflammatory cytokine, interleukin-6 (IL-6), are secreted from MPTCs with increasing numbers of fibrogenic HSCs. Reprinted with permission from Davidson *et al.*, Integr. Biol. **9**(8), 662. Copyright 2017 Oxford University Press.

MPCCs containing PHHs can be infected with both HCV^37^ and HBV^38^ and are able to replicate infectious virions for ∼3 weeks [Fig. 1(a)]. However, for HBV, the use of broad-spectrum Janus Kinase (JAK) inhibitors is necessary to attenuate the innate immune response and allow hepatocytes to sufficiently replicate the virus. In contrast, randomly distributed cocultures of the same two cell types (PHHs and 3T3-J2 fibroblasts) were not able to chronically support HCV or HBV replication in the aforementioned studies, potentially due to an incomplete polarization and lower functionality of the hepatocytes than in MPCCs. However, another group has recently shown that randomly distributed PHH/3T3-J2 cocultures created from several PHH donors are able to support HBV infection for up 30 days without the use of the JAK inhibitor as long as the virus was purified in a specific way and a proprietary commercially available culture medium was utilized;^39^ it remains unclear in a direct comparison using these specialized reagents whether MPCCs can continue to outperform the randomly distributed cocultures. Regardless, across all the above studies, stabilized PHHs cocultured with 3T3-J2 fibroblasts were shown to be a robust model for evaluating the interplay between drug metabolism, toxicity, and efficacy since all three aspects are critical for drug effectiveness in the clinic.

The PHH/3T3-J2 cocultures, in either MPCC or randomly distributed formats, require the use of polyethylene glycol (PEG) at high multiplicity of infection (MOI) to effectively infect the PHHs with HBV; such a protocol does not mimic the natural infection of PHHs with HBV *in vivo* via cell receptors such as sodium taurocholate cotransporting peptide (NTCP). Furthermore, the use of PHHs alone in culture platforms does not allow modeling of infected PHH cross talk with NPCs in the liver involved in inflammation and fibrosis. To mitigate such limitations, the “LiverChip” containing PHH/KC aggregates adhered to collagen-coated and perfused polycarbonate microchannels was shown to support infection and replication of HBV from both cell culture-derived and patient-derived HBV sources for up to 40 days across multiple donors without the use of PEG and at MOIs that were 10 K-fold lower than other tested models (spheroids and randomly distributed PHH/3T3-J2 cocultures); furthermore, HBV-infected PHH/KC aggregates in the LiverChip were useful to evaluate the effects of direct acting antiviral drugs on secretion of HBV DNA and antigens in a nondestructive manner [Fig. 1(b)].^40^ The ability of LiverChip to sustain clinically relevant HBV infection is likely due to a combination of 3D spheroidal architecture and fluid perfusion (can form gradients of oxygen, nutrients, and hormones as in the liver sinusoid) since neither 2D PHH cultures nor static PHH spheroids reached the same levels of liver function or infection as the LiverChip. In particular, the inclusion of KCs in this chip allowed investigation of how HBV could potentially evade the immune response in the liver and establish chronic infection. However, similar to other advanced liver models, the virus did not spread from infected cells to neighboring uninfected cells at the low MOIs, which suggests that additional host factors or cell populations may be involved in HBV spread as *in vivo* (>80% of the human liver becomes infected).

While PHHs can now be successfully infected with HCV and HBV in different engineered platforms as discussed above, these cells do not allow modeling of genotype-phenotype relationships across many donor backgrounds due to a restricted supply of donor organs rejected for transplantation into patients. To mitigate this limitation, iHep monocultures have been shown to be successfully infected with HCV^41^ and HBV^38,42^ which enables the investigation of the effects of donor genotype and host genes on infection efficiency, propagation, and resistance to drug therapies. However, 2D iHep monocultures are also known to lose drug metabolism capacity over time,^36^ which limits their use for evaluating the critical interplay between the metabolism, toxicity, and efficacy (i.e., inhibition of viral load) of novel drug therapies and their combinations. We have recently shown that the MPCCs created using iHeps (instead of PHHs) show higher drug metabolism capacity than iHep monocultures that can be chronically infected with both HCV and HBV for ∼3 weeks *in vitro* and can serve as a robust platform for drug screening (manuscript in preparation). We anticipate that further progress with utilization of iHeps in engineered platforms for HCV/HBV infection will enable patient-specific drug testing for these diseases. However, two key issues with induced pluripotent stem cells (iPSCs) technology will need to be addressed including continuous optimization of culture protocols to further mature iHeps toward the adult PHH phenotype and differentiation of iPSCs into liver NPCs to adequately model interaction of multiple liver cell types with the same donor background.^43,44^

Some useful observations from the aforementioned liver model examples are that neither biomimicry of liver architecture nor liver-derived NPC types from even the same species are necessary for enabling high levels of PHH (or iHep) functions and infection with HCV/HBV *in vitro*. Such observations suggest that liverlike microenvironmental signals (soluble and insoluble) are the most important for stabilizing the hepatic phenotype. In particular, neither MPCCs nor the LiverChip mimic the architecture of the liver; yet, both platforms are able to keep hepatocytes highly functional for several weeks *in vitro*. Furthermore, MPCCs use 3T3-J2 fibroblasts, which likely mimic a developmental program that crosses the species barrier to provide differentiation signals to the neighboring hepatocytes. The ability to use different engineered culture formats for long-term PHH/NPC cocultures provides the end-user a high level of flexibility to vary the throughput and cellular complexity *in vitro* to test the hypotheses being posed.

### Nonalcoholic fatty liver disease

NAFLD is on an epidemic rise (∼1 in 4 individuals in the US), is a major risk factor for type 2 diabetes mellitus,^45^ and can progress to inflammation, fibrosis, and liver cancer, which is a virtually untreatable disease.^46^ Animal models of NAFLD suffer from significant differences in NAFLD^47^ and drug metabolism^48^ pathways as compared to humans; thus, human liver models are essential for preclinical drug development. Conventional 2D PHH monocultures have been shown to become steatotic (fatty) upon treatment with mixtures of saturated and unsaturated fatty acids toward mimicking the early stages of NAFLD.^49^ However, since these cultures display a rapid decline in the activities of drug metabolism enzymes, their use for screening novel drug therapies to alleviate hepatic steatosis is challenging. Furthermore, conventional 2D PHH monocultures with or without a Matrigel™ overlay (a.k.a. sandwich cultures) spontaneously lose sensitivity to pancreatic hormones, insulin, and glucagon,^50^ which precludes their use to determine how overnutritional stimuli and ensuing steatosis cause insulin resistance in PHHs. Notably, hepatocytic insulin resistance, which is characterized by the inability of hepatocytes to regulate glucose metabolism in response to insulin (i.e., glucose uptake and downregulation of gluconeogenesis in the presence of insulin are impaired), is a major outcome of the NAFLD disease spectrum.^51^

In contrast to the above-mentioned limitations with conventional PHH monocultures, MPCCs were shown to retain *in vivo-*like responsiveness to insulin and glucagon for 3+ weeks as assessed via the dynamics of glycogen storage and gluconeogenesis.^50^ In a follow-up study, MPCCs exposed to a hyperglycemic culture medium for 3 weeks developed steatosis and became resistant to insulin-mediated suppression of gluconeogenesis concomitantly, while other measured liver functions (i.e., CYP activities, albumin secretion, and urea synthesis) were not affected [Fig. 1(c)].^52^ Further treating the steatotic MPCCs with the antidiabetic drug, metformin, significantly reduced gluconeogenesis. Interestingly, MPCCs treated with a hypoglycemic culture medium increased CYP activity, which can have implications for patients with type 2 diabetes mellitus, who experience hypoglycemia due to side effects of specific drug therapies.

Similar to the above MPCC platform, the LiverChip containing perfused PHHs was incubated for 14 days with excess nutrition, specifically palmitic and oleic free fatty acids (FFA).^53^ While the FFAs were not toxic to PHHs, several genes associated with NAFLD were increased, whereas CYP activities were reduced. Metformin therapy reduced the steatosis in PHHs relative to vehicle-treated control cultures. Even static PHH spheroids generated via ultralow attachment plates have been shown to mimic the early stages of NAFLD [Fig. 1(d)].^54^ Specifically, PHH spheroids incubated with pathophysiological concentrations of FFAs, glucose, fructose, and high levels of insulin became steatotic and concomitantly displayed enhanced expression of genes associated with *denovo* lipogenesis and insulin resistance. These symptoms could be alleviated by either removing the pathophysiological stimuli or treatment with antisteatotic compounds.

While phenotypically stabilized PHHs can be used to investigate steatosis and insulin resistance as discussed above, the later stages of NAFLD, called nonalcoholic steatohepatitis (NASH), cause inflammation and fibrosis in the liver, which are mediated by liver NPCs. For instance, with progressing disease, HSCs become activated into myofibroblasts that secrete proinflammatory cytokines and deposit excessive collagen, which is a hallmark of fibrosis.^46^ Reversing such fibrosis using pharmaceuticals can potentially halt the progression of NASH into cirrhosis and HCC. To model the interactions between HSCs and stabilized PHHs, a micropatterned triculture (MPTC) platform was developed in which (a) micropatterned PHHs were functionally stabilized using the 3T3-J2 fibroblasts (as in MPCCs described above) and (b) the PHH phenotype was modulated by culturing activated (fibrogenic) HSCs within the fibroblast monolayer at physiologically relevant ratios with PHHs; such a triculture configuration was used since the HSCs were not able to stabilize the PHH phenotype to the same magnitude and longevity as the fibroblasts.^34^ While albumin and urea secretions were relatively similar in MPTCs and MPCCs (suggesting well-differentiated PHHs), over the course of 2 weeks, increasing HSC numbers within MPTCs downregulated hepatic CYP and drug transporter activities, caused hepatic steatosis independent of overnutritional stimuli, and enhanced the secretion of proinflammatory cytokines, which are effects also observed clinically in patients suffering from early stages of NASH/fibrosis [Fig. 1(e)].^55,56^ Importantly, inhibition of nicotinamide adenine dinucleotide phosphate oxidase (NADPH oxidase) and/or activation of farnesoid X receptor (FXR) using clinically relevant drugs, GKT137831 and obeticholic acid (OCA), respectively, alleviated hepatic dysfunctions in MPTCs at nontoxic concentrations, thereby suggesting MPTC utility for screening the efficacy and toxicity of anti-NASH/fibrosis drugs.

In another example of an engineered NASH human liver model by HemoShear Therapeutics, Inc., PHHs were cultured in a collagen gel on one surface of a polycarbonate membrane, while a mixture of HSCs and macrophages was cultured on the other surface of the membrane.^57^ The NPC compartment was subsequently subjected to hemodynamic flow using a cone-and-plate viscometer, while the hepatic compartment was subjected to continuous perfusion to recapitulate interstitial-like flow patterns. When this platform was exposed for 10 days to a lipotoxic milieu (high insulin and glucose and FFAs), PHHs accumulated lipids, increased glucose output, and displayed reduced insulin sensitivity.^57^ Furthermore, inflammatory markers were secreted at higher levels, and HSCs displayed increased activation as assessed via the staining of alpha-smooth muscle actin. Importantly, transcriptomic and lipidomic data obtained *in vitro* correlated to some extent with those obtained from human liver NASH biopsies. Treating the *in vitro* diseased cocultures with OCA led to improvements in the lipidomic signature and a reduction in inflammatory and fibrotic secreted factors.

Finally, a bioprinted human liver spheroid (centimeter scale) that contains a compartment of PHHs next to a NPC compartment containing HSCs and endothelial cells housed in a 24-well transwell format^58^ can also be induced toward a steatotic state when treated with excess FFAs; furthermore, HSCs expressed higher alpha-smooth muscle actin staining, a marker of fibrosis, in such bioprinted livers.^59^

It is now clear through the aforementioned pioneering studies that engineered human liver models can be coaxed into a steatotic phenotype via treatment with overnutrition stimuli (e.g., glucose, fructose, FFAs, and high insulin levels) for a few weeks. Inclusion of activated HSCs into PHH-based models has shown that these cells secrete inflammatory cytokines, secrete collagen, and express markers reminiscent of the early stages of fibrosis in NAFLD. However, the ability to transdifferentiate quiescent HSCs (vitamin A storing cells in the liver) to an activated phenotype via incubation with excess nutritional stimuli has not been achieved in human systems since commercially available HSCs have already become myofibroblasts due to their expansion onto stiff tissue culture plastic. Isolating fresh quiescent HSCs for every experiment is unpractical for routine drug screening; thus, the ability to design substrates that can revert activated HSCs to a more quiescent phenotype is desirable. High-throughput ECM microarrays provide an opportunity to determine the optimal ECM (protein composition and substrate stiffness) and soluble factor microenvironment that can induce quiescent states in human liver NPCs as well as enable optimal functions in PHHs. In such microarrays, cells are seeded onto printed spots of biomolecules that include adhesive components to promote localized cell adhesion as well as combinations of other factors to stimulate or measure cellular processes.^60–64^ Furthermore, it is currently not possible to mimic the more severe stages of NAFLD *in vitro*, namely, cirrhosis and HCC. Nonetheless, we anticipate that even human liver models of the early stages of NAFLD, which have been already developed, will prove to be highly useful to develop drug therapies that target different molecular aspects of this disease; certainly, Takeda pharmaceuticals is leading the way via their substantial partnership with HemoShear Therapeutics to discover novel drugs for NAFLD using their perfused human liver platform. Finally, additional efforts are also needed to better understand the processes that mediate the progression from NAFLD to HCC. In particular, NAFLD-associated HCC in the absence of cirrhosis has been identified in some cases,^65,66^ suggesting that a linear progression through worsening stages of cirrhosis may not be a requirement for HCC. The application of engineered *in vitro* culture models could enable the types of systematic studies required to evaluate the connections between the genetic and microenvironmental changes occurring during NAFLD and HCC tumorigenesis.

A key advantage of a 3D liver model for NAFLD/NASH studies is the ability to determine reorganization of cells and ECM with fibrosis progression. However, while self-assembled spheroids enable 3D cell-cell and cell-ECM interactions, it is difficult to form structurally stable spheroids with >50% of PHH donors/lots,^67^ potentially due to variable ECM secretion rates across PHH donor cells. Encapsulating cells or spheroids in hydrogels can mitigate the above-mentioned limitation, but the choice of the hydrogel type is important. While bioinert alginate^68^ and poly(ethylene glycol) (PEG)^69^ have been used to encapsulate hepatocytes, neither material type allows the cells to remodel the ECM as in fibrosis progression. Thus, natural ECM containing collagens is preferable for modeling liver fibrosis. While 3D bioprinting can be used to embed cells in ECM hydrogel “inks” and create on-demand architectures, this is an expensive and low-throughput process requiring an unsustainably large number of expensive and limited PHHs for applications in drug development.^58^ Furthermore, large (>500 *μ*m) hydrogels pose significant diffusion limitations for oxygen and nutrients to cells in the construct's core;^70^ this limitation can be mitigated by miniaturizing the hydrogel scaffolds to ∼100–300 *μ*m. Such miniaturized cell-laden hydrogels can be created using microfluidic droplet generators, which typically use fluorocarbon oil with surfactant to create aqueous emulsions containing unpolymerized ECM mixed with cells.^71,72^ We have recently utilized high-throughput droplet microfluidics to fabricate collagen-based 3D human liver microtissues containing PHHs and 3T3-J2 fibroblasts that displayed 6+ weeks of functions, which were up to 10-fold higher in levels than conventional self-assembled spheroids and bulk collagen hydrogels with encapsulated cells.^176^ We anticipate that such a platform will be useful to study reorganization of cell-cell and cell-ECM interactions in fibrosis induced by not only NAFLD but also HCV and HBV infections.

Gradients of oxygen and hormones play important roles in the induction of differential functions in hepatocytes along the length of the sinusoid, a phenomenon termed “zonation”;^73,74^ as many as 50% of liver genes are found to be zonated.^75^ In addition to its key role in normal liver physiology, steatosis resulting from NAFLD is typically localized in the perivenous zone with low O_2_ levels,^76,77^ and is more often associated with hepatocyte injury and advanced fibrosis in human NAFLD liver biopsies.^78^ While aspects of liver zonation, especially those related to drug-induced cell responses (e.g., toxicity and induction of CYP enzymes), have been modeled *in vitro* in several studies,^79–83^ application to modeling NAFLD is lacking and thus presents an opportunity for further research in this space.

### Liver cancer

#### Current strategies and limitations

Chronic liver diseases such as NAFLD or chronic hepatitis infection are associated with an increased risk for the primary liver cancer termed hepatocellular carcinoma (HCC).^84–87^ HCC accounts for approximately 70%–80% of all liver cancers and is the second leading cause of cancer-related death globally.^88,89^ Additional types of liver cancers include cholangiocellular carcinoma (CCC),^90^ a malignancy of the liver bile ducts, and hepatoblastoma,^91^ which is the most common pediatric malignant tumor of the liver. For HCC, depending on the stage at presentation, treatments range from resection and ablation for the early stage, transarterial chemoembolization for the intermediate stage, and kinase inhibitors and supportive care for advanced stage disease.^92^ Numerous ongoing research studies are focused on the development and application of new therapeutic strategies including checkpoint inhibitors and procedures, such as selective internal radiotherapy, which could be used throughout the distinct disease stages.^93–95^ For pediatric hepatoblastoma, although early stage tumors can often be cured by surgery alone without chemotherapy, all other stages usually require aggressive cisplatin/doxorubicin-based chemotherapy together with surgical resection and in some cases liver transplantation.^96^

A number of challenges exist for the development of new and more effective treatments for liver cancers. Typically, liver cancer surveillance and diagnosis is achieved through a combination of abdominal ultrasonographic imaging, biopsy analysis, and serum biomarker assessment.^97^ The sensitivity of imaging and biomarker-based methods is often a limitation.^98^ Also, heterogeneity within a liver tumor can enhance the possibility that biopsy samples do not accurately represent the collective characteristics of the tumor.^99^ Further, although several common driver mutations have been identified in liver cancers, there is an increasing appreciation for the significant interpatient variability often observed. For example, in hepatoblastoma, despite the commonalities in beta-catenin mutational status observed across a majority of hepatoblastoma patients, the presentation of hepatoblastoma is highly diverse, with the tumors typically classified based on morphological patterns.^100^ Interpatient variability can also greatly influence the overall response to specific therapies. For instance, variations in the response rate of HCC patients to the kinase inhibitor sorafenib have been observed, which has led to a number of studies aimed at identifying the factors underlying these variations and toward the establishment of molecular predictors of treatment response.^101–103^

One major limitation that greatly influences not only the development of new drug treatments but also the overall understanding of liver cancer progression and heterogeneity is the lack of appropriate *in vitro* cell culture models. To better recapitulate patient genetics and interpatient variations, numerous efforts have focused on the generation of culture models that utilize cells directly from tumor biopsies or patient-derived xenograft (PDX) systems, as opposed to cultures using strictly HCC cell lines. However, independent of the cell source, conventional culture configurations often do not accurately mimic the microenvironmental signals present within liver tumors. Accordingly, many of the engineered culture approaches introduced earlier in this perspective have been applied toward studies aimed at investigating liver cancer processes and for evaluating therapeutic responsiveness as part of the preclinical development phase. In the next sections, we will highlight a number of these efforts with a focus on recent advances in the areas of three-dimensional (3D) liver cancer microenvironments, microfluidic devices, and patient-specific organoid systems.

##### 3D liver cancer microenvironments

For a wide range of tumor cell types, 3D cell cultures have been suggested to better represent i*n vivo* microenvironments, including more *in vivo*-like gene expression profiles and drug responses.^104–107^ One common approach for evaluating tumor cells in a 3D context is the establishment of 3D aggregates, often referred to as multicellular spheroids. Enhanced drug resistance within 3D spheroids, compared to standard 2D cultures, has been observed for numerous cancer cell lines and treatment protocols.^108,109^ In particular, spheroidal tumor cell cultures have been demonstrated to exhibit a number of signaling pathway alterations such as changes in epidermal growth factor family, mitogen-activated protein kinase, and protein kinase B (AKT)-mammalian target of rapamycin (mTOR) signaling that can broadly influence tumor cell phenotype and drug sensitivities.^110–112^ For liver tumor cell spheroids, the “age”/culture time of the spheroidal culture, ranging from 1 to 18 days, was demonstrated to influence drug diffusivity and toxicity, with increased drug resistance at the later stages of culture.^113^ Further, proteomic analysis of liver tumor spheroids cultured in a rotating wall vessel bioreactor highlights the dynamic changes in protein expression patterns that occur over two weeks of culture.^114^ Collectively, such results suggest that microenvironmental signals including cell-cell and cell-ECM interactions, as well as microenvironmental pH or hypoxia, likely evolve during the culture period. As a means to generate spheroidal tumor cell cultures with well-defined cell numbers and drug diffusion characteristics, several approaches including hanging drop cultures and microfabricated microwells have been employed. For example, 3D spheroids of the HCC cell line Huh7 were formed using a hanging drop method combined with rotary culture, and it was demonstrated that large spheroids (diameter ∼ 3 mm) exhibited apoptosis and increased expression of hypoxia-inducible factor-1-alpha (HIF-1alpha) in the center of the spheroid and an overall increase in phosphorylated extracellular-signal-regulated kinase (ERK) compared to smaller spheroids.^115^ Microwell substrates for generating defined multicellular tumor spheroids have been produced using a variety of strategies including the molding of agarose hydrogel scaffolds,^116^ the molding of collagen coated PDMS arrays compatible with molecular imaging techniques,^117^ and the fabrication of networked concave PDMS microwell structures.^118^ Each of these approaches was utilized for examining drug cytotoxicity, with the goal of integrating 3D spheroid culture with drug screening. Microwells have also been combined with micropillar structures toward the development of a modular 2-chip platform for testing cancer therapeutics.^119,120^ In this strategy, tumor cell aggregates are encapsulated within 30 nl alginate hydrogels on top of an array of micropillar structures using a spotting process. The inversion of the micropillar chip, and insertion of the micropillar-resident cells into the adjacent microwell substrate chip containing panels of drug solutions, can then facilitate high-throughput drug efficacy testing.

The introduction of additional cell types for the generation of coculture spheroids has also been explored. For instance, the incorporation of human umbilical vein endothelial cells (HUVECs) into large Huh7 spheroids was shown to promote Huh7 proliferation and an increased expression of cancer stem cell markers.^115^ The introduction of a human hepatic stellate cell line (LX2) into spheroidal cultures generated using both liver tumor cell lines and primary HCC cells identified an effect of stellate cells on increasing spheroid compactness and decreasing drug sensitivity.^121^ Recently, HCC cell lines specifically derived from HBV-infected patients were utilized for drug response experiments incorporating coculture spheroids containing HUVECS, stellate cells, or human fibroblasts.^122^ In addition, stellate cell conditioning media have been shown to promote proliferation of human HCC cells within spheroidal cultures.^123^ Stellate cell production of factors such as ECM protein collagen type I,^121^ and cytokine IL-6,^124^ has been determined to be relevant as part of the stellate cell-mediated alterations in liver tumor cell responses. Overall, since stellate cell activation and proliferation are correlated with the presence and progression of chronic liver diseases, which in turn are associated with an increased liver cancer risk, the continuous investigation of stellate cell-tumor cell intercellular interactions is highly warranted.

**FIG. 2.**
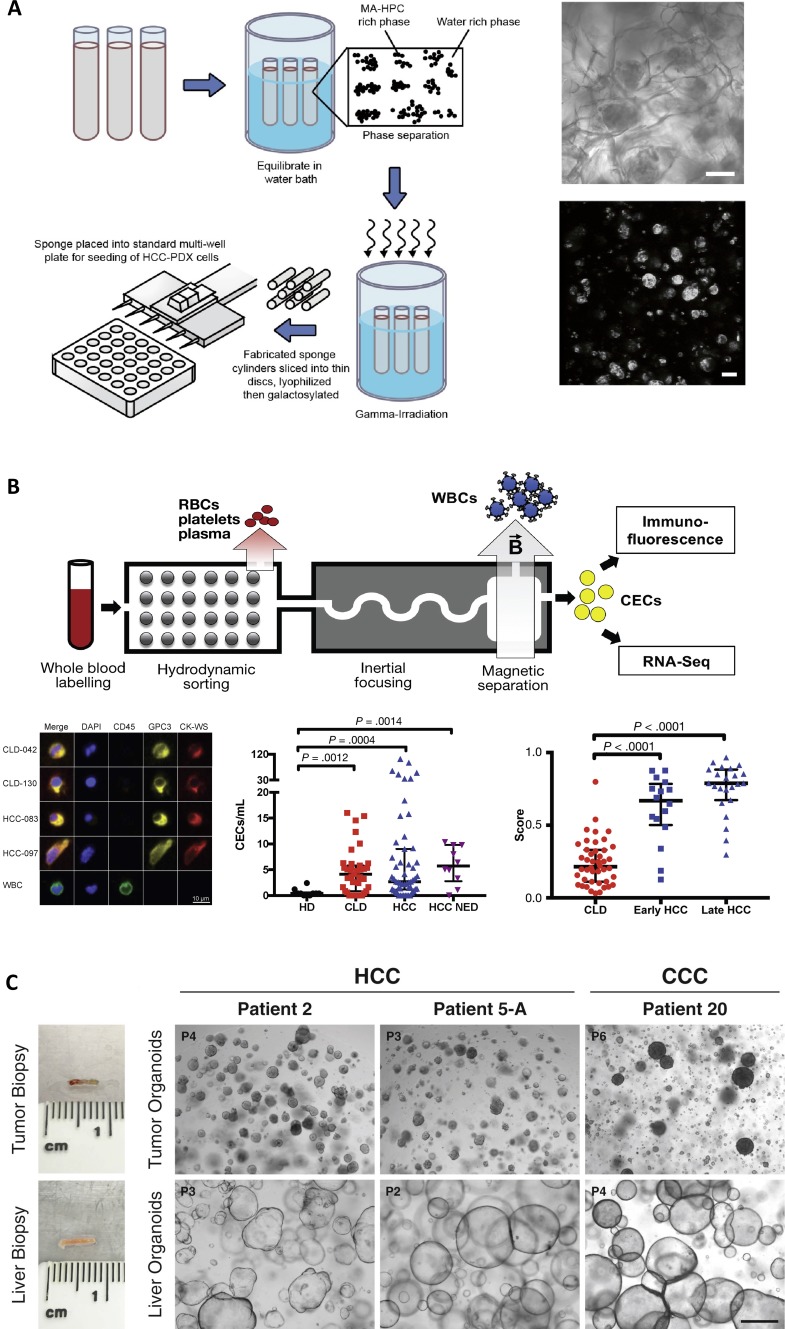
Engineered culture systems and devices for liver cancer. (a) Three-dimensional scaffold culture model for hepatocellular carcinoma (HCC) incorporating patient-derived xenograft cells, figure adapted from Ref. 127. Left: Schematic of the process for fabricating a macroporous hydrogel sponge from hydroxypropyl cellulose (HPC) using photocrosslinking following the introduction of methacrylate (MA) groups. Right: Brightfield (top) and phalloidin-based actin staining (bottom) of HCC cells cultured in a representative MA-HPC scaffold (scale bars = 100 *μ*m). Reprinted with permission from Fong *et al.*, Biomaterials **159**, 229 (2018). Copyright 2018 Elsevier. (b) Chip-based approach for the detection and analysis of circulating epithelial cells (CECs) in patients with liver cancer, figure adapted from Ref. 158. Top: Schematic of the microfluidic device (iChip) process, which separates CECs from hematopoietic cells for subsequent staining or sequencing analysis. Bottom left: CECs collected using the iChip process were stained with DAPI (blue), anti-CD45 (green) to detect hematopoietic cells, as well as anti-glypican-3 (GPC3, yellow) and antiwide spectrum cytokeratin (CK-WS, red), which are both epithelial markers. iChip-enriched samples from two chronic liver disease without HCC (CLD) patients and two HCC patients are shown. A control white blood cell (WBC) is also shown as a comparison. Bottom middle: Quantification of CECs using immunofluorescence of the iChip-processed sample, from healthy donors (HDs), chronic liver disease patients without HCC (CLD), HCC patients (HCC), and treated HCC patients with no evidence of malignant disease (NED). Bottom right: HCC score derived from gene signature analysis of cells post-iChip separation from CLD, early HCC, and late HCC patients. Reprinted with permission from Bhan *et al.,* Gastroenterology **155**(6), 2016 (2018). Copyright 2018 Elsevier. (c) Organoid cultures derived from human liver cancer patients; figure adapted from Ref. 169. Organoid cultures were established from needle biopsies from liver cancer patients. Left: Representative biopsy pieces of tumor tissue and paired nontumor liver tissue used for organoid generation shown on the right. Right: Brightfield images of tumor and paired nontumor liver tissue organoids from three different patients, including two HCC patients and one cholangiocellular carcinoma (CCC) patient (scale bar = 500 *μ*m). Reprinted with permission from Nuciforo *et al.*, Cell Rep. **24**(5), 1363 (2018). Copyright 2018 Elsevier.

The encapsulation of liver tumor cells (as either multicellular spheroids or dispersed cell suspensions) within biomaterial scaffolds has also been broadly used for the study of tumor cell behavior in 3D. For instance, coculture spheroids containing the human liver tumor cell line HepG2 together with murine fibroblasts were encapsulated within collagen gels, and the presence of both the fibroblasts and the collagen gel contributed to increased resistance to doxorubicin treatment.^125^ In another work, HepG2 and Hep3B cell lines were examined following seeding within the commercial AlgiMatrix™ culture system (Thermo-Fisher), which is a porous alginate scaffold.^126^ Within the AlgiMatrix™ scaffold, cells increased expression of the cancer stem cell marker EpCAM, exhibited morphogenetic patterns consistent with acinar formation, and were more resistant to chemotherapeutic agents compared to cells in standard 2D culture. In addition, building on the studies using liver tumor cell lines, recent efforts have begun to evaluate the possibility of incorporating PDX-derived cells within biomaterial-based *in vitro* 3D culture models. For example, 14 distinct PDX lines were derived from HCC patients and cultured within a 3D macroporous cellulosic sponge scaffold [Fig. 2(a)].^127^ Whole exome and RNA sequencing were performed following *in vitro* scaffold culture, and an overall positive correlation was observed with the *in vivo* PDX models. Further, the scaffold cultures were compatible with cytotoxicity analysis, suggesting that this *in vitro* culture approach could be utilized for preclinical drug testing.

Toward the further advancement of 3D scaffold systems, the assessment of the effects of scaffold biochemical and biomechanical properties on liver tumor cell behavior is another point of emphasis. In particular, analogous to studies examining the effects of fibrotic liver microenvironments on hepatocyte functions, it is hypothesized that modular hydrogel systems that exhibit defined material characteristics could similarly provide insights into microenvironmental regulation of liver tumor cell processes. In studies using the encapsulation of the HepG2 cell line in hybrid PEG-collagen hydrogels, it was demonstrated that cells cultured in softer hydrogels formed spheroids with a malignant phenotype, while cells in stiffer hydrogels formed more compact structures with suppressed malignancy.^128^ Although these hybrid hydrogels were selected such that they exhibited a fairly uniform mesh size across the distinct stiffnesses, continuous endeavors in the field aim to further decouple the effects of tissue stiffness, porosity, and biomaterial/ECM composition on cell responses. For example, complementary efforts using 2D polyacrylamide substrates with defined elastic moduli have demonstrated that increased stiffness can upregulate the expression of osteopontin,^129^ and cancer stem cell markers,^130^ in HCC cell lines. Huh7.5 cells encapsulated within PEG-diacrylate hydrogels exhibited increased proliferation, as well as increased hepatocellular functions such as albumin secretion and CYP450 expression, in hydrogels with relatively low elastic modulus (∼0.1 kPa), and the modification of the hydrogels with fibrinogen further modulated encapsulated spheroid growth.^131^ In the 3D PEG-collagen hydrogel system, scaffold softening through the treatment with exogenous matrix metalloproteinase (MMP)-1 was shown to enhance HepG2 proliferation, decrease the expression of E-cadherin, and increase the sensitivity to radiation exposure.^132^ In addition to synthetic hydrogels, recent studies have examined the efficacy of decellularized rodent,^133,134^ porcine,^135,136^ and human^137^ liver tissues as the basis for 3D culture approaches aimed at better representing the complement of ECM factors present *in vivo*. In order to directly evaluate the ECM composition and the presence of other key signals within typical liver tumor microenvironments, a broad range of studies have employed a combination of animal models together with the direct assessment of patient samples.^138^ For future efforts, the integration of proteomics-based analysis of patient tissues,^139^ including comparisons to associated chronic liver diseases such as fibrosis,^140^ could help to identify additional microenvironmental cues for integration into biomaterial scaffold culture models.

#### Microfluidic chip-based analysis of cancer progression

Microfabricated devices, such as microfluidic chips, have been applied toward the study of a wide range of cancer types including liver cancer. Overall, the modularity of microfluidic device design has enabled the investigation of numerous aspects of cancer progression including vascularization, metastasis, immune interactions, and tissue mechanics.^141–144^ For instance, a microfluidic platform was developed and applied toward the evaluation of liver cancer cell line migration in response to physical cues such as confinement.^145^ A microfluidic approach has also been employed for single cell capture and subsequent growth of multicellular spheroids,^146^ and the integration of liver tumor cell/stellate cell coculture spheroids with drug treatment experiments.^147^ Multiorgan on chip systems incorporating liver cell types have also been explored.^148,149^ For example, a microfluidic system containing both HepG2 cells and a human colon cancer cell line was established for the measurement of colon cell migration toward the distinct liver culture chamber, as a model of colon tumor cell metastasis.^150^ Broadly, such multiorgan platforms could enable unique investigations into the pharmacokinetics of liver cancer therapies and the overall effects of metabolite and drug transport on tumor responses.

In addition, a recent series of investigations have highlighted the capability of microfluidic-based coculture models for analyzing interactions between liver tumor cells and hematopoietic cells, which underlie the potential effectiveness of immunotherapies. In particular, in one approach, HepG2 spheroids expressing a component of the HBV genotype D envelope protein were encapsulated within a collagen gel located in the central channel of the device.^151^ HBV-specific T cells were introduced into the outer channels with or without monocytes, and following coculture, it was determined that the monocytes reduced T cell-mediated cytotoxicity, which is consistent with the suggested physiologic function of monocytes within tumor microenvironments.^152^ Notably, this immunosuppressive effect was specific to the 3D microfluidic coculture, as it was not observed in parallel experiments using standard 2D cocultures. Further, using a similar device design that incorporated either dispersed or aggregated HepG2 cells within collagen gels, together with engineered T cells in adjacent channels, the co-operative effects of cytokines and oxygen tension on tumor cell killing were examined.^153^ These studies demonstrated that the presence of inflammatory cytokines, IFN-gamma and TNF-alpha, enhanced the capability of T cells to lyse tumor cell multicellular aggregates, and for the dispersed tumor cells, T cell-mediated killing was decreased in 2% O_2_ compared to the 20% O_2_ condition.

Microfluidic devices have also been utilized in the setting of cancer diagnostics, including most notably an approach for detecting and enriching circulating tumor cells as part of the assessment of liquid biopsies.^154^ Circulating tumor cells have been identified in patients with HCC,^155,156^ and recent efforts have aimed to pair circulating tumor cell enrichment with a variety of downstream analysis strategies. For example, HCC patient blood samples were enriched for circulating tumor cells using a microfluidic chip based on the capture and positive selection of cells expressing asialoglycoprotein receptor (ASGPR), which is selectively expressed by hepatic cell types.^157^ These enriched cells were then utilized to establish 3D spheroid cultures and were tested for sensitivity to chemotherapeutic drugs. In another set of studies, a microfluidic device termed the iChip was applied toward the enrichment of circulating HCC cells^158,159^ [Fig. 2(b)]. In this platform, patient blood first undergoes a size-based separation that removes red blood cells, platelets, and plasma, followed by magnetic depletion of white blood cells using magnetic beads conjugated with antibodies to hematopoietic markers (CD45 and CD66b).^160^ Cells enriched from HCC patients using this device were demonstrated to be compatible with RNA-based digital polymerase chain reaction (PCR) measurements for the quantitative analysis of gene expression.^159^ In addition, the iChip platform was also recently applied toward the parallel evaluation of both circulating tumor cells from HCC patients and circulating epithelial cells from chronic liver disease patients without HCC.^158^ Accordingly, this type of approach for analyzing liquid biopsies could have great utility toward the tracking of chronic liver disease (e.g., fibrosis) severity and for liver cancer surveillance.

#### Patient-specific tumor organoids

Building on the knowledge gained from healthy adult stem cell organoids, numerous recent efforts have focused on organoids as potential model systems for cancer research.^161^ Long-term organoid cultures have been initiated directly from patient-derived tumor tissues. For instance, primary and metastatic breast cancer organoid culture lines have been demonstrated to exhibit strong correlations with the original tumors with regard to numerous criteria including DNA copy number variations, hormone receptor status, and HER2 status.^162^ Patient-derived organoids from gastroesophageal and colorectal cancer patients were demonstrated to enable molecular profiling toward the prediction of responses to anticancer therapeutics, which matched well with patient responses in clinical trials.^163^ Further, in recent studies investigating colon cancer, organoid cultures originated with healthy stem cells, and gene editing strategies were utilized to investigate genetic perturbations underlying colorectal tumorigenesis.^164,165^

Long-term organoid cultures have also been established directly from patient-derived tumor tissue in the context of liver cancers. For example, primary liver cancer organoids were generated from HCC, CCC, and combined HCC/CCC tumors.^166^ Similar to other stem cell-based organoid culture approaches, these tissue samples were suspended within a basement membranelike hydrogel and the culture medium consisted of an optimized cocktail of growth factors and small molecules. These patient-derived liver cancer organoids maintained histological features and gene expression characteristics of the original tumors and also supported drug screening, which led to the identification of an ERK inhibitor as a promising therapeutic candidate based on growth inhibitory effects observed on some of the patient organoid lines. For the analysis of CCC, defined genetic modifications in normal human cholangiocyte organoids revealed the important role of the deubiquitinating enzyme BAP1 in the CCC malignant phenotype.^167^ In another work, the generation of multiple organoid lines, taken from distinct regions of the same liver tumors, provided unique insights into intratumor heterogeneity and how such heterogeneity can influence drug efficacy.^168^ Recent efforts have also demonstrated the capability to generate tumor organoids from needle biopsies taken from HCC and CCC tumors, as well as from comparison nontumor liver biopsy samples from the same patients [Fig. 2(c)].^169^ In these studies, organoids derived from tumor biopsies formed compact spheroids, while the nontumor samples grew as larger cystic structures, and collectively, the tumor organoids maintained the genetic alterations identified within the source tumors. In addition, the organoid cultures were compatible with the assessment of drug responses, with the HCC-derived organoids exhibiting variable sensitives to the drug sorafenib.

Moving forward, continuous efforts will likely focus on ways to further integrate organoid culture models with high-throughput drug screening. For instance, recent studies examining colorectal cancer have implemented various automation tools for pairing the culture of patient-derived spheroidal aggregates with the evaluation of >2400 drugs.^170^ Modification of the culture configurations may also enable increased throughput. For example, the establishment of tumor organoids as rings around the exterior of the culture wells can help to facilitate the types of automated sample handling required for large-scale screens.^171^ In addition, despite the extensive insights into tumor genetics that have been provided by organoid systems, much less is known about how extracellular signals modulate organoid processes. For instance, murine CCC organoids that show a reproducible ductlike phenotype *in vitro* have been demonstrated to exhibit broader differentiation plasticity following subsequent transplantation into recipient mice,^172^ which underscores the involvement of tumor cell-extrinsic factors. Notably, by interfacing with strategies for engineering 3D cultures discussed earlier in this review, tumor organoid cultures could ultimately be utilized as a tool to investigate the regulatory roles of factors within the tumor microenvironment. Such efforts have recently been pursued for epithelial stem cell organoids, in which defined hydrogel materials have provided important clues into how the ECM composition, mechanical stiffness, and material degradation influence organoid growth and morphogenesis.^173–175^

## CONCLUSION

Engineered human liver platforms with varying throughputs and technological complexities are now available to investigate liver disease and drug outcomes at different stages of preclinical drug development. The use of these platforms will continue to increase with continuous participation from the pharmaceutical industry and regulatory agencies in validating and adopting commercially available platforms. Furthermore, these platforms can be used for elucidating structure-function relationships in liver physiology and disease. Overall, we anticipate that the increased use of these optimized *in vitro* platforms should reduce the overall cost of drug development, reduce the reliance on *in vivo* animal studies, and accelerate the market availability of novel drugs. The beneficiaries of such advances will be patients who will have faster and more cost-effective access to highly efficacious and safe drug therapies.
